# Using Health Information Technology to Reach Patients in Underserved Communities: A Pilot Study to Help Close the Gap with Health Disparities

**DOI:** 10.5539/gjhs.v8n6p86

**Published:** 2015-10-20

**Authors:** Mark H. Ryan, Jonathan Yoder, Sharon K. Flores, Jason Soh, Allison A. Vanderbilt

**Affiliations:** 1 Department of Family Medicine and Population Health, International/Inner City/Rural Preceptorship (I2CRP), Virginia Commonwealth University, Richmond, Virginia, USA; 2 Department of Family Medicine, University of Massachusetts Memorial Medical Center, Fitchburg, MA, USA; 3 Department of Family Medicine and Population Health, Faculty Services Coordinator and Global Health Program Coordinator, School of Medicine, Virginia Commonwealth University, Richmond, Virginia, USA; 4 Department of Pediatrics, Oregon Health and Science University, Portland, Oregon, USA; 5 Department of Family Medicine, College of Medicine and Life Sciences at the University of Toledo, Toledo, OH, USA

**Keywords:** technology, health disparities, underserved populations, medical education

## Abstract

**Introduction:**

In the current era of medical education and curriculum reform, medical schools across the United States are launching innovative approaches to teaching students in order to improve patient outcomes and increase patient safety. One such innovation is the use information technology (IT) that can be used to disseminate health information, especially for patients with limited access to care. Strategies for using health IT to enhance communication between providers and patients in low-income communities can be incorporated into undergraduate medical education (UME) curriculum.

**Methods:**

A pilot study was conducted to determine if IT could serve as an effective means of communication with patients at a free clinic where 100% of the patients are uninsured; the clinic is located in an urban setting and primarily serves Latinos, the working poor, and the homeless. An anonymous survey was administered to patients to assess rates of IT ownership, general IT use, and IT use for health and medical information.

**Results:**

The majority of study participants owned a cell phone (92%); one-third used their cell phone to access health or medical information (38%). Most study participants reported using the Internet (72%), and two-thirds had used the Internet to obtain health and medical information (64%).

**Conclusion:**

Given the difficulties faced by low income and medically underserved communities in accessing healthcare services, the use of IT tools may improve their’ access to health information in ways that could enhance patient knowledge and self-management, and perhaps positively impact health outcomes. Therefore, it is essential to incorporate use of IT tools in training for medical students and residents to enhance communication with patients in underserved communities.

## 1. Introduction

The utilization of information technology (IT) is widespread among adult Americans. Approximately 76% of adults own a computer and as of early 2013, 85% of adults used the Internet (Smith, 2010; Pew Research Center’s Internet & American Life Project Spring Tracking Survey, 2013). Cellphone ownership and use is even more common. In the United States, 91% of adults own a cellphone, and 63% use their cellphones to access the Internet or “go online.” (Duggan & Smith, 2013) Furthermore, 56% of adults own a “Smartphone”, 93% of whom use their cellphones to access the Internet (Duggan & Smith, 2013; Smith, 2013). Among all adults who go online, 72% use social networking sites (Brenner & Smith, 2013). This widespread use of IT has the potential to impact the dissemination of health information.

Medical information and health are commonly searched and identified using IT. By 2012, 72% of adult Internet users (approximately 35% of US adults) looked online for health information (Fox & Duggan, 2013). Also, nearly one-third of Smartphone owners used these devices to look for health information (Fox, 2012). Even though individuals with one or more chronic diseases were less likely than their healthy peers to have Internet access, 62% were able to access online information (Fox, 2011). However, while more Americans are going online to find health and medical information, healthcare providers are consistently recognized as the most influential source of information for medical decisions (Fox & Duggan, 2013; Couper et al., 2010). Healthcare providers have responded to their patients’ use of IT by going online themselves; responding to patients’ questions posted on the Internet in forums such as blogs, chat rooms, or via email with their patients; and by communication via secure patient portals.

Although IT use relative to the general population is lower among adults with an annual income of less than #30,000 and among adults with a less than high school education, their overall use of IT is still high. Among adults with an annual income of less than #30,000, 59% own a computer, 76% use the Internet, 55% use the Internet on their cellphones, and 75% of the Internet users use social networking sites (Smith, 2010; Pew Research Center’s Internet & American Life Project Spring Tracking Survey, 2013; Duggan & Smith, 2013; Brenner & Smith, 2013). Among adults with a less than high school education, 42% own a computer, 59% use the Internet, 51% use the Internet on their cellphones, and 67% of the Internet users use social networking sites (Smith, 2010; Pew Research Center’s Internet & American Life Project Spring Tracking Survey, 2013; Duggan & Smith, 2013; Brenner & Smith, 2013). National surveys have shown that ownership and use of IT among Latinos is substantial, even when considering Latinos who are foreign-born, with an income of less than #30,000 per year, or with less than high school education (Lopez, Gonzalez-Barrera, & Patten, 2013); nearly half of all adult Latinos looked online for health information (Fox & Duggan, 2013). Similarly, a study of homeless patients seen in an emergency department revealed no significant difference in their use of IT compared to non-homeless patients (Post et al., 2013).

At the international level, there has been investigation in how mobile phone tools can enhance the work of community health workers in low-income countries (DeRenzi et al., 2011). While the use of mobile health (mHealth), described as “the use of portable electronic devices with software applications to provide health services and manage patient information”, appears to have significant potential to enhance healthcare in underserved settings but there is little available information demonstrating an improvement in clinical outcomes (Källander et al., 2013; Peiris et al., 2014). With the large-scale ownership and usage of cell phones in low-income countries (Swahn, Braunstein, & Kasirye, 2014; Aker & Mbiti, 2015), however, it is clear that an opportunity exists to leverage this technology for health care if a viable model can be developed.

According to the 2010 National Healthcare Disparities Report, disparities related to race, ethnicity, and socioeconomic status exist within the American health care system (Gibbons et al., 2011). Because of the high prevalence of IT use among minority Americans, a thorough examination of technology use among these populations may reveal opportunities to use IT to address existing healthcare disparities (Christopher Gibbons, 2011). A recent review article examining the benefits of social media for health communication found that the use of social media increased health information access to younger people, ethnic minorities, and lower socioeconomic groups (Moorhead et al., 2013). The extent of health IT use is not known among low income individuals in the Richmond, Virginia area, Therefore, the purpose of this study was to address the following research question: Are health information technology usage patterns among patients at CrossOver Healthcare Ministry, located in downtown Richmond, Virginia, similar to those found in similar populations surveyed elsewhere in the United States?

## 2. Methods

This cross-sectional study received prior approval from the Virginia Commonwealth University Institutional Review Board. The study was conducted from February-March 2014 at CrossOver Healthcare Ministry, a free clinic that primarily serves medically uninsured Spanish-speaking Latinos, the working poor, and the homeless, all of whom are under 200% of the federal poverty level. Study participants were adult patients of Cross Over Healthcare Ministry’s downtown Richmond site.

A survey tool was developed for the study and used to collect data from participants. The survey was anonymous and borrowed elements from online surveys previously published by the PEW Research Center (Pew Research Center’s Internet & American Life Project Spring Tracking Survey, 2013). The survey used simple language and was available in both English and Spanish. The survey included questions regarding: demographics (age, race, and primary language), access to healthcare (excellent, good, fair, and poor), health (excellent, good, fair, and poor), chronic medical condition (yes, no), IT ownership, general use of IT, and IT use for health and medical information. IT devices included desktop computer, laptop, netbook, basic cell phone, smartphone, or tablet computer.

Social work student interns provided the survey to patients in the clinic waiting room after check-in. The interns asked patients if they were willing to fill out the survey and handed the survey to them if they agreed, but the interns did not directly administer the survey. Study participants were informed that the survey was for research purposes, that it was not mandatory, that it was anonymous, and that completion of the survey would not affect the provision of healthcare. Participants were instructed to complete the survey while they were in the waiting room (or in the exam room while they waited for the physician, if more time was required) and return it to the intern or front desk staff.

Demographic, Cronbach’s alpha, and frequency data were analyzed using Stata 10.1 (StataCorp, College Station, Texas) and IBM SPSS Statistics for Windows, Version 22.0. Armonk, NY: IBM Corp.

## 3. Results

The Cronbach’s alpha for the survey instrument was a 0.79 demonstrating internal consistency among participants who completed the survey. Overall, 39 surveys were collected and analyzed, 14 (36%) of which were in English and 25 (64%) were in Spanish. The majority of the study participants were ages 25-64 (70%), reported having at least one chronic health condition (67%), gave a subjective health rating of good or fair (85%), rated their access to healthcare as excellent or good (72%), and owned an IT device (92%) Table 1.

**Table 1:** Demographics

		English survey N=14		Spanish survey N=25		Total surveys N=39	

		n (%^0^)					
Age							
	18-24	0	(0)	2	(8)	2	(5)
	25-34	0	(0)	8	(32)	8	(21)
	35-44	2	(14)	4	(16)	6	(15)
	45-54	7	(50)	1	(4)	8	(21)
	55-64	3	(21)	2	(8)	5	(13)
	64+	1	(7)	1	(4)	2	(5)
Race							
	White	1	(7)	0	(0)	1	(3)
	Hispanic	0	(0)	18	(72)	18	(46)
	African American	10	(71)	0	(0)	10	(26)
	Other	2	(14)	0	(0)	2	(5)
Chronic health condition							
	No	1	(7)	8	(32)	9	(23)
	Yes	12	(86)	14	(56)	26	(67)
Rated health							
	Excellent	0	(0)	1	(4)	1	(3)
	Good	6	(43)	13	(52)	19	(49)
	Fair	7	(50)	7	(28)	14	(36)
	Poor	0	(0)	1	(4)	1	(3)
Rated access to healthcare							
	Excellent	5	(36)	6	(24)	11	(28)
	Good	6	(43)	11	(44)	17	(44)
	Fair	1	(7)	5	(20)	6	(15)
	Poor	1	(7)	1	(4)	2	(5)
Ownership of IT devices^1^							
	No	1	(7)	1	(4)	2	(5)
	Yes	13	(93)	23	(92)	36	(92)

0 May not add up to 100% due to missing data;

1 Information Technology (IT) devices include any of the following; desktop computer, laptop, netbook, basic cell phone, smartphone, or tablet computer.

The majority of study participants owned a cell phone (92%) Table 2. Approximately half of study participants used a cell phone to send or receive e-mail (59%). Approximately one- third used a cell phone to send or receive text messages (38%), access the Internet (31%), use social media applications (38%), or access health or medical information (38%).

**Table 2:** Cell phone ownership and use

	English survey N=14		Spanish survey N=25		Total surveys N=39	

	n (%^0^)					
Cell phone ownership							
No	1	(7)	1	(4)	2	(5)
Yes	13	(93)	23	(92)	36	(92)
Send or receive text messages							
No	4	(29)	8	(32)	12	(31)
Yes	10	(71)	5	(20)	15	(38)
Send or receive e-mail							
No	8	(57)	4	(16)	12	(31)
Yes	6	(43)	17	(68)	23	(59)
Access the internet							
No	7	(50)	8	(32)	15	(38)
Yes	7	(50)	5	(20)	12	(31)
Use social media applications							
No	9	(64)	7	(28)	16	(41)
Yes	5	(36)	10	(40)	15	(38)
Participate in a video call/chat							
No	13	(93)	11	(44)	24	(62)
Yes	1	(7)	2	(8)	3	(8)
Health Information^1^							
No	9	(64)	14	(56)	23	(59)
Yes	5	(36)	10	(40)	15	(38)

0 May not add up to 100% due to missing data;

1 Using cell phone to look up health or medical information.

The majority of study participants reported ever using the Internet (72%) Table 3. Approximately half of study participants used the Internet to send or receive e-mail (46%). Approximately one-third used the Internet to get news online (31%), research a product or service (41%), or use a social networking site (38%). A lower number of participants reported using the Internet to access Twitter or other services to share updates (10%).

**Table 3:** Internet use

		English survey N=14		Spanish survey N=25		Total surveys N=39	

		n (%^0^)					
Any internet use	No	4	(29)	6	(24)	10	(26)
	Yes	10	(71)	18	(72)	28	(72)
Send or receive e-mail	No	4	(29)	16	(64)	20	(51)
	Yes	9	(64)	9	(36)	18	(46)
Get news online	No	7	(50)	14	(56)	21	(54)
	Yes	7	(50)	5	(20)	12	(31)
Research a product or service	No	6	(43)	11	(44)	17	(44)
	Yes	8	(57)	8	(32)	16	(41)
Use social networking sites	No	7	(50)	11	(44)	18	(46)
	Yes	7	(50)	8	(32)	15	(38)
Use Twitter or other service^1^	No	11	(79)	13	(52)	24	(62)
	Yes	2	(14)	2	(8)	4	(10)

0 May not add up to 100% due to missing data;

1 Use Twitter or other service to share updates about yourself or others.

More than half of all study participants used the Internet for health and medical information (64%) Table 4. Among study participants who use the Internet, the vast majority reported ever using the Internet for health and medical information (89%). Among the study participants who completed the Spanish survey, 68% reported ever using the Internet for health and medical information. Approximately, one-third of study participants reported searching online about health or medical issues (41%), posting health-related material online (28%), or using a social networking site for health-related activity (38%).

**Table 4:** Internet use for health and medical information

	English survey N=14		Spanish survey N=25		Total surveys N=39	

	n (%^0^)					
Any internet use for health and medical information						
No	1	(7)	0	(0)	1	(3)
Yes	8	(57)	17	(68)	25	(64)
Search online about certain health or medical issues^1^						
No	7	(50)	4	(16)	11	(28)
Yes	7	(50)	9	(36)	16	(41)
Online activity regarding health^2^						
No	1	(7)	1	(4)	2	(5)
Yes	7	(50)	14	(56)	21	(54)
Post health-related material online						
No	1	(7)	2	(8)	3	(8)
Yes	1	(7)	10	(40)	11	(28)
Use social networking site for health related activity^3^						
No	1	(7)	1	(4)	2	(5)
Yes	4	(29)	11	(44)	15	(38)

0 May not add up to 100% due to missing data;

1 Search online about certain health or medical issues including any of the following; specific disease or medical problem, certain medical treatment or procedure, doctors or health professionals, hospitals or medical facilities, health insurance, environmental hazards, pregnancy/child birth, end of life decisions, long-term care for elderly or disabled person, food safety/recalls, drug safety/recalls, chronic pain management, medical test results, or other health issue;

2 Signed up to receive email updates or alerts about health or medical issues, read someone else’s commentary or experience about health or medical issues on an online news group/website/blog, watched an online video about health or medical issues, gone online to find others who might have health concerns similar to your own, tracked your weight/diet/exercise routine(s), or tracked any other health indicator(s) or symptom(s) online;

3 Get health info, start or join a health-related group, follow friend’s personal health updates, raise money or draw attention to a health-related cause, or remember of memorialize others who suffer from a certain health condition.

Approximately, half of the study participants reported non-specific health-related online activity, which was defined as follows: ever signing up to receive email updates or alerts about health or medical issues; reading someone else’s commentary or experience about health or medical issues on an online news group/website/blog; watching an online video about health or medical issues; going online to find others who might have similar health concerns; tracking weight/diet/exercise routine(s) and/or tracking any other health indicator(s) or symptom(s) online.

Finally, the vast majority of study participants owned an IT device (92%). The majority of study participants owned a cell phone (92%) and one-third reported ever using their cell phone to access health or medical information (38%). The majority of study participants reported ever using the Internet (72%) and two-thirds used the Internet for health and medical information (64%). These results are reflective of health IT use among adult Americans (64% versus 72%) generally.^6^ Also, 68% of study participants who completed the Spanish survey reported using the Internet for health and medical information, a higher percentage than that found in national surveys showing that nearly half of all adult Latinos looked online for health information (Fox & Duggan, 2013).

## 4. Limitations

In addition to small sample size, only one site from one organization was used in this study and thus the data may not be representative of all low-income patients.

## 5. Discussion

A recent report by the Commonwealth Fund (Broderick & Haque, 2015) indicates that although cellphones may be a valuable tool in enhancing health care for vulnerable populations, only a small proportion of safety net clinics are using cellphones in patient care. Most of the clinics that responded their survey indicated they use cellphones to provide information (such as appointment or immunization reminders), as opposed to facilitating patient engagement. The most common limitations to providing cell phone-based services to patients were limited funding and lack of human and IT resources.

Thus, there is a great disparity in health and healthcare access, but little disparity in IT use between the general population and Latinos, the working poor, and the homeless, suggesting a window of opportunity through which IT may be used to help improve health and healthcare access for low income populations. The result of this small pilot study suggests that IT ownership and use of health IT at CrossOver Healthcare Ministry is comparable to the general adult American population. Further assessment of health IT use and attitudes towards health IT may help guide future projects related to the use of health IT to improve access to healthcare.

The clinical possibilities that arise from this information are both clear and important. Providing patient information via online resources may allow clinicians to provide patients with information about their own health concerns as well as broader public health issues. Rather than searching the Internet generally, patients may be able access pertinent information provided (or curated) by their own clinician. Such an approach could guide patients to reliable information and ensure that they are making health care decisions on the basis of good evidence-based information.

Given the difficulties faced by low income and medically underserved communities (such as literacy and transportation), using online and mobile IT tools may serve to enhance their access to healthcare information in ways that could enhance patient knowledge and self-management, and perhaps positively impact health outcomes. There is a strong body of research demonstrating that patients who rate highly on patient-activation scales have lower healthcare costs and improved health statuses (Mosen et al., 2007; Hibbard & Greene, 2013; Parchman, Zeber, & Palmar, 2010; Greene & Hibbard, 2012). If healthcare providers use patient-centered communication accessed via tools and approaches that are convenient and familiar to patients, it may be possible to enhance patients’ ability to take care of their illnesses outside of the medical setting.

As medical offices move to the use of online patient portals via electronic health records (EHR), secure digital communication with patients will increase in importance and may allow patients more effective and convenient communication with their physicians’ offices. Although communication via EHR portals is different from general online communication, text messaging, and social media, the convergence of these trends points towards the importance of training medical students to become effective communicators in the digital arena. Whether they are posting blogs about important healthcare topics, texting patients about test results or follow-up appointments, or communicating with patients via a secure EHR portal, medical students must be taught the skills to do so effectively and safely.

The importance of training students in digital communication skills extends into the areas of program review and accreditation. The Liaison Committee on Medical Education (LCME) Standard 7 (Curricular Content) includes Standard 7.8: “The faculty of a medical school ensures that the medical curriculum includes specific instruction in communication skills as they relate to communication with patients and their families, colleagues, and other health professionals.” (Liaison Committee on Medical Education, 2015) In most medical school curricula, this instruction is focused on face-to-face or, at most, telephone-based communication. The authors could not identify any medical school that includes meaningful training on digital communication as part of their curriculum. As communication continues to move in the direction of using mobile and digital IT tools, it is increasingly important to prepare our students with needed skills in those areas.

The AGCME includes “Interpersonal and Communication Skills” among its current six Core Competencies: “Residents must be able to demonstrate interpersonal and communication skills that result in effective information exchange and teaming with patients, patients’ families, and professional associates.” (Accreditation Council on Graduate Medical Education, 2015) As the ACGME moves towards its new accreditation system based on milestones developed out of these competencies, and as medical schools are responsible for developing core entrustable professional activities (EPAs) for “milestone 0” graduates from medical schools, preparing medical students to communicate digitally with peers, patients, and the public will become increasingly important.

As medicine moves further into the digital arena where patients seek to engage with providers asynchronously and via IT devices, it is a necessary step for medical schools to prepare graduates with the knowledge, skills, and attitudes to communicate effectively in these ways. Given the ready access to IT devices and the multiple roles in which patients seek to access healthcare, there are many opportunities to explore enhancing patient knowledge and engagement and, perhaps, improve health for low income and marginalized populations.

## 6. Conclusion

In this pilot survey, access to, and use of, mobile devices and online resources were comparable among patients of a free clinic in an urban setting. Though by definition low income (<200% federal poverty level), most patients owned a cell phone, and the majority had used Internet-based resources to access health information. More than one-third of patients who responded to the survey had used a cell phone to access healthcare information. These results are reflective of the national trends in the use of mobile devices and online resources (Pew Research Center’s Internet & American Life Project Spring Tracking Survey, 2013).

The fact that an increasing number of Americans are looking online for health information creates an opportunity to engage patients and provide information where it is already sought. The reality that most Americans are have access to IT devices such as cell phones creates an opportunity to communicate with patients in a manner that might be both more efficient and more convenient for physicians and patients alike. The results of this pilot study are important as they show that, at least in the setting surveyed, low-income patients are just as likely to have access to mobile devices and to use online resources as other Americans.
